# The impact of dialysis on critically ill elderly patients with acute kidney injury: an analysis by propensity score matching

**DOI:** 10.1590/2175-8239-JBN-2018-0058

**Published:** 2018-08-02

**Authors:** Flávio Teles, Renata Oliveira Santos, Helder Marx Almeida de Moura Lima, Rodrigo Peixoto Campos, Eline Calumby Teixeira, Ananda Camilla de Andrade Alves, André Falcão Pedrosa Costa, Jorge Artur Peçanha de Miranda Coelho

**Affiliations:** 1 Universidade Estadual de Ciências da Saúde de Alagoas, Maceió, AL, Brasil.; 2 Universidade Federal de Alagoas, Maceió, AL, Brasil.; 3 Santa Casa de Misericórdia de Maceió, Maceió, AL, Brasil.

**Keywords:** Renal Insufficiency, Chronic, Renal Dialysis, Intensive Care Units, Aged, Insuficiência Renal Crônica, Diálise Renal, Unidades de Terapia Intensiva, Idoso

## Abstract

**Introduction:**

Aging is a global phenomenon. Recent forecasts indicate that Brazil will be the sixth country in population of elderly individuals in 2020. The incidence of acute kidney injury (AKI) among the elderly varies, but studies have indicated that older individuals are more prone to developing AKI and have higher mortality rates than the general population with renal disease. The impact of dialysis in elderly patients with AKI - and critically ill individuals with multiple dysfunctions - has been discussed for years. Evidence indicates that for this group of patients dialysis does not positively impact survival and, in some situations, it might even accelerate death. This study investigated a population of elderly individuals with AKI seen in intensive care units to assess, through Propensity Score Matching, the impact dialysis has had for them.

**Methods:**

Data from the charts of patients aged 60 years or older seen at the intensive care unit of a general hospital between January 2012 and December 2014 and diagnosed with AKI were collected.

**Results:**

The study included 329 patients with a mean age of 75.4 ± 9.3 years. Ischemic AKI was the most prevalent disease (54.7%) and 28.9% of the patients needed dialysis. No difference was seen in the death rates of dialysis and non-dialysis patients aged 70+ years.

**Conclusions:**

The data suggested that dialysis did not seem to impact the death rates of critically ill patients with AKI aged 70+ years.

## RESUMO

**Introdução:**

O crescimento da população idosa é um fenômeno mundial. Projeções recentes demonstram que o Brasil será o sexto país do mundo em número de idosos no ano de 2020. A incidência de injúria renal aguda (IRA) nos idosos varia de acordo com a população estudada, mas vários estudos têm sugerido que os idosos são mais suscetíveis ao desenvolvimento de IRA e apresentam maior mortalidade que a população geral. Há anos se discute o real impacto da instituição da diálise em pacientes idosos com IRA, principalmente em pacientes críticos com múltiplas disfunções. Há evidências de que o início da diálise nesses indivíduos não tenha impacto positivo na sobrevida e, em algumas situações, até acelere o óbito. O objetivo deste estudo foi analisar uma população de idosos que desenvolveu IRA em unidades de terapia intensiva e avaliar, através do *Propensity Score Matching*, o impacto da diálise nesses indivíduos.

**Métodos:**

Foram coletados dados de prontuários de idosos acima dos 60 anos, internados na UTI de um hospital geral, de janeiro de 2012 a dezembro de 2014, e que evoluíram com IRA.

**Resultados:**

Foram incluídos 329 pacientes, com idade média de 75,4 ± 9,3 anos. A IRA isquêmica foi a mais prevalente (54,7%), e 28,9% necessitaram de diálise. Nos indivíduos acima de 70 anos, não foi observada diferença na mortalidade entre os pacientes que realizaram ou não diálise.

**Conclusões:**

Esses dados sugerem que a diálise parece não apresentar impacto sobre a mortalidade de pacientes críticos com IRA, acima de 70 anos.

## INTRODUCTION

Life expectancy has grown steadily all over the world. Moreover, the advancement of medical science has enabled elderly patients with severe disease to survive for longer. This scenario, nonetheless, begs the question as to whether advanced life support truly impacts the progression of elderly patients on intensive care or if it simply introduces additional suffering to the final period of one's life.^1^^,^ 2 ^,^ 3 ^,^ 4

Dialysis is one of the therapies prescribed to support the lives of patients in critical condition by enabling the establishment of a more suitable metabolic and nutritional state to individuals with acute kidney injury (AKI).

However, dialysis entails risks and complications such as the ones related to implanting venous catheters, hemodynamic instability, changes in antibiotic levels, and bleeding on account of heparin administration. In recent years, the impact of dialysis on the progression of patients with AKI has been discussed. Particular attention has been devoted to the elderly, a group in which organ dysfunction and comorbidities are seen more frequently.^2^^,^ 3 ^,^ 5

AKI is a common finding in hospital settings strongly associated with increased mortality. Prevalence may be as high as 50% in critically ill patients.^6^ Advanced age is a known risk factor for AKI. Feest *et al*. reported up to eight-fold increases in the prevalence of AKI in patients aged 60+ years.^7^

In addition to increased prevalence, evidence indicates that advanced age is a risk factor for death and permanent loss of renal function requiring the prescription of chronic dialysis.^1^^,^ 2 ^,^ 8 ^,^ 9

In this population, the decision to start dialysis is based on general clinical findings such as signs and symptoms of AKI, and may not take into account the risks inherent to the procedure, the desire of the patient and of his or her family, and overall quality of end of life.^3^^,^ 4

Despite the progress seen with the use of hemodialysis in intensive care settings, the impact of dialysis on elderly patients with multiple comorbidities or organ failure (prescribed vasoactive drugs or mechanical ventilation) has been discussed for years.^7^^,^ 8 Apparently, dialysis does not improve patient survival and, in some situations, may even accelerate death or increase end-of-life distress.^4^^,^ 5 However, there is little evidence on the matter in the literature.

This study aimed to assess the impact of dialysis in the survival of elderly patients with AKI on intensive care.

## METHODS

This retrospective cohort study included data collected from the electronic charts of patients diagnosed with AKI during hospitalization at the intensive care unit of the Santa Casa de Misericórdia de Maceió, AL, Brazil, from January 2012 to December 2014. The Santa Casa is a reference hospital located in the capital city of the State of Alagoas, Brazil. The nephrologists present in the hospital are involved in the everyday care and assessment of renal patients.

Patients with 60+ years of age diagnosed with AKI according to the KDIGO criteria^10^ were included in the study. Three hundred and eighty-two charts were reviewed, and 329 met the inclusion criteria. Patients without sufficient clinical or workup data were excluded. The study was initiated after the approval of the Research Ethics Committee of the Universidade Estadual de Ciências da Saúde de Alagoas (UNCISAL) (protocol no.: 62798216.3.0000.5011).

The patients enrolled in the study were characterized according to age, sex, etiology of AKI (ischemic, nephrotoxic, obstructive or mixed) and other variables such as occurrence of septic shock (acute circulatory failure caused by infection), oliguria (urine output below 400ml in 24 hours), need for mechanical ventilation (MV) and vasoactive drugs (VAD), use of diuretics, dialysis, and hospital death (at the ICU or in the hospital wards after discharge from the ICU). Comorbidities were assessed based on the Charlson Comorbidity Index. The number of days between the first alteration in creatinine levels and assessment by a nephrologist was also analyzed (ΔT nephro). The following lab variables were assessed: creatinine, urea, potassium, and complete blood count.

Baseline creatinine was set as the lowest level found during hospitalization. Only traditional hemodialysis (HD) was offered, with session times ranging from two to four hours, blood and dialysate flow rates set at 300 and 500 ml/min respectively, using polysulfone capillary filters with a surface area of 1.8 m^2^ and an ultrafiltration coefficient of 7.5 ml/h/mmHg. Dialysis was interrupted in cases of severe hemodynamic instability after the start of the procedure. Indications for dialysis included: azotemia with uremic symptoms (usually with urea levels > 150 mg/dL), oliguria refractory to diuretics (urine output < 400 ml in 24 hours), hyperkalemia refractory to drug therapy (K+ > 6.5 mmol/L), hypervolemia and metabolic acidosis (pH < 7.2 and serum bicarbonate < 16 mEq/L in arterial blood). The patients were divided into age ranges (60 to 70; 70 to 80; and 80+ years) for analysis.

Numerical variables were expressed as mean values ± standard deviation (SD) or median values and interquartile ranges depending on whether they followed a normal distribution. The associations between continuous variables were measured through Student's t-test or ANOVA, while categorical variables were analyzed with the chi-squared test. Variables eliciting significant differences in univariate analysis were tested with logistic regression for independent associations with death (Enter method). In order to study the impact of dialysis on death, the patients were divided into two groups based on whether they underwent dialysis and were matched for the main variables that might have an effect on death, such as use of vasoactive drugs, mechanical ventilation, oliguria, KDIGO, and others. Statistical method Propensity Score Matching was used. The level of significance was set at α = 0,05 with a 95% confidence interval. Statistical analysis was performed on SPSS (version 23).

## RESULTS

The study included 329 individuals. General, clinical, and workup data are described on Table 1. The patients had a mean age of 75.4 ± 9.3 years and 168 (51.1%) were males. One hundred and five (31.9%) were aged 60-70 years; 121 (36.8%) were aged 70-80 years; and 103 (31.3%) were aged 80+ years. Eighty-two (24.9%) patients had septic shock, 202 (61.4%) had oliguria, and 194 (59%) took diuretics. Vasoactive drugs were prescribed to 159 (48.4%) and mechanical ventilation to 236 (71.7%). The mean ΔT nephro was 3.8 ± 6.8 days. Dialysis was offered to 95 patients (28.9%). The mean Charlson Comorbidity Index was 6.9 ± 2.1.

**Table 1:** General sample data (n = 329)

Variables	
Age	75.46 ± 9.3
Age ranges	
60-70 years	105 (31.9%)
70-80 years	121 (36.8%)
80+ years	103 (31.3%)
Male sex	168 (51.1%)
Etiology of AKI	
Ischemic	180 (54.7%)
Nephrotoxic	13 (4%)
Obstructive	36 (10.9%)
Mixed	100 (30.4%)
Plasma K+	5.46 ± 2.0
Hb	8.33 ± 2.0
Leukocyte count	19.300 [27.450-12.550]
Platelet count	111.000 [172.000-63.000]
Oliguria	202 (61.4%)
Use of diuretics	194 (59%)
Dialysis	95 (28.9%)
Septic shock	82 (24.9%)
Mechanical ventilation	236 (71.7%)
Vasoactive drugs	159 (48.4%)
Charlson Comorbidity Index	6.92 ± 2.14
Death	203 (61.7%)
KDIGO	
KDIGO 1	85 (25.8%)
KDIGO 2	86 (26.1%)
KDIGO 3	158 (48%)

Data expressed as mean values ± standard deviation or median and interquartile ranges.

In regards to severity of AKI, 85 (25.8%) patients were categorized as KDIGO 1; 86 (26.1%) as KDIGO 2; and 158 (48%) as KDIGO 3. In terms of etiology of AKI, 180 (54.7%) had ischemic AKI; 36 (10.9%) had AKI caused by obstruction; 13 (4%) had AKI on account of nephrotoxicity; and 100 (30.4%) had AKI with multifactorial etiology. Overall mortality of the sample was 61.7% (203 patients).

When the patients were divided based on age ranges, the individuals aged 70-80 and 80+ years were found to share many traits. The differences observed in some variables were more pronounced when the comparison was made against the patients in to the younger age group (60-70 years, see Table 2). For example, the Chalrson Comorbidity Index and need for mechanical ventilation were higher among patients aged 70-80 and 80+ years when compared to the individuals in the 60-70 year age group. The individuals aged 60-70 years had significantly higher baseline creatinine levels than the patients aged 70-80 and 80+ years (*p* = 0.05). The same happened to peak creatinine. In terms of etiology of AKI, patients aged 70+ years had more ischemic AKI, while individuals aged 60-70 years had more multifactorial AKI (*p* = 0.01). The distribution of KDIGO categories and mortality were similar between the three age ranges. Fewer patients in the group aged 80+ years were prescribed dialysis, regardless of their KDIGO classification (only 18.4% of the group *vs*. 36.2% of the individuals aged 60-70 years and 31.4% of the patients aged 70-80 years; *p* = 0.01). The indication of dialysis was analyzed vis-à-vis the KDIGO classification. In KDIGO 3 individuals, in which dialysis is more likely to be indicated, fewer patients aged 80+ years were prescribed dialysis (only 28% *vs*. 46.2% of the patients aged 60-70 years and 41.8% of the patients aged 70-80 years; *p* = 0.06). The death rate of KDIGO 3 patients aged 70+ years requiring mechanical ventilation and vasoactive drugs was 93.5% (29). Of the 29 patients who did not survive, 55.2% were on dialysis. The death rate of KDIGO 3 individuals aged 80+ years requiring mechanical ventilation and vasoactive drugs was 100% (26). Thirty-eight percent of the 26 patients underwent dialysis.

**Table 2:** Distribution of variables in accordance with age ranges

	60-70 (n = 105)	70-80 (n = 121)	80 + (n = 103)	*p*
Male sex	55 (52.4%)	68 (56.2%)	45 (43.7%)	0.16
Septic shock	30 (28.6%)	31 (25.6%)	21 (20.4%)	0.37
Vasoactive drugs	58 (55.2%)	50 (41.3%)	51 (49.5%)	0.10
Mechanical ventilation	60 (61%)	90 (74.4%)	82 (79.6%)	0.009
Cr (baseline)	1.40 ± 0.79	1.22 ± 0.65	1.19 ± 0.58	0.05
Cr (max)	4.53 ± 3.1	3.88 ± 2.1	3.53 ± 2.0	0.01
Cr (nephro)	3.34 ± 2.4	2.89 ± 1.5	2.63 ± 1.6	0.02
Urea (max)	180 ± 90.7	172 ± 80.8	178 ± 71.5	0.76
Plasma K^+^	5.3 ± 1.5	5.6 ± 2.7	5.4 ± 1.1	0.48
Hemoglobin	8.1 ± 2.1	8.1 ± 2.1	8.7 ± 1.9	0.04
Leukocyte count	18.700[25.700-11.350]	19.600[27.750-12.650]	19.200[27.300-14.000]	0.92
Platelet count	121.000 [207.500-50.000]	98.000 [164.500-61.800]	118.000 [164.000-75.000]	0.06
Etiology of AKI				0.01
Ischemic	45 (42.9%)	77 (63.6%)	58 (56.3%)	
Nephrotoxic	4 (3.8%)	4 (3.3%)	5 (4.9%)	
Obstructive	9 (8.6%)	15 (12.4%)	12 (11.7%)	
Mixed	47 (44.8%)	25 (20.7%	28 (27.2%)	
KDIGO				0.90
KDIGO 1	27 (25.7%)	32 (26.4%)	26 (25.2%)	
KDIGO 2	30 (28.6%)	28 (23.1%)	28 (27.2%)	
KDIGO 3	48 (45.7%)	61 (50.4%)	49 (47.6%)	
Charlson	5.52 ± 1.78	7.36 ± 2.21	7.82 ± 1.65	0.0001
Delta Nephro	3.74 ± 6.8	4.38 ± 7.4	3.28 ± 6.4	0.49
Oliguria	67 (63.8%)	70 (57.9%)	65 (63.1%)	0.60
Diuretics	68 (65.4%)	65 (53.7%)	61 (59.2%)	0.20
Dialysis	38 (36.2%)	38 (31.4%)	19 (18.4%)	0.01
Death	59 (56.2%)	76 (62.8%)	68 (66%)	0.33

Table 3 shows the distribution of variables in accordance with the occurrence of death. Dialysis was more frequent among non-survivors (35.5% *vs*. 18.3% of survivors; *p* = 0.001). A greater proportion of the patients who died had oliguria (74.9% *vs*. 39.7% of the survivors; *p* = 0.0001), septic shock (36.9% *vs*. 5.6%; p = 0.0001), took diuretics (63.9% *vs*. 51.6%; *p* = 0.02), was on vasoactive drugs (67% *vs*. 18.3%; *p* = 0.0001), and required mechanical ventilation (88.7% *vs*. 44.4%; *p* = 0.0001). The other variables did not vary significantly in relation to mortality.

**Table 3:** Distribution of variables in accordance with mortality

	Non-survivors (n = 203)	Survivors (n = 126)	*p*
Male sex	98 (48.3%)	70 (55.6%)	0.19
Oliguria	152 (74.9%)	50 (39.7%)	0.0001
Diuretics	129 (63.9%)	65 (51.6%)	0.02
Charlson	7.02 ± 2.1	6.76 ± 2.2	0.28
Mechanical ventilation	180 (88.7%)	56 (44.4%)	0.0001
Vasoactive drugs	136 (67%)	23 (18.3%)	0.0001
Septic shock	75(36.9%)	7 (5.6%)	0.0001
Delta Nephro	4.1 ± 7.5	3.3 ± 5.7	0.31
Hemoglobin	8.0 ± 2.0	8.7 ± 2.0	0.003
Platelet count	92.000 [152.000-50.000]	151.500[192.000-93.750]	0.0001
Leukocyte count	21.700 [30.900-15.700]	15.550[22.075-9.900]	0.01
Peak urea	194 ± 87	148 ± 60	0.0001
Baseline Cr	1.28 ± 0.73	1.25 ± 0.61	0.67
Peak Cr	3.93 ± 2.08	4.07 ± 3.06	0.63
Plasma K^+^	5.5 ± 2.3	5.3 ± 1.3	0.38
KDIGO			0.55
KDIGO 1	49 (24.1%)	36 (28.6%)	
KDIGO 2	52 (25.6%)	34 (27%)	
KDIGO 3	102 (50.2%)	56 (44.4%)	
Dialysis	72 (35.5%)	23 (18.3%)	0.001

Data expressed as mean values ± standard deviation or median and interquartile ranges or absolute numbers and proportions.

On account of the similarities between the groups aged 70-80 and 80+ years, the logistic regression model and Propensity Score Matching (PSM) were applied to individuals aged 70+ years. In the logistic regression model (Table 4), the variables independently correlated with death were septic shock (OR = 3.97; CI = 1.15, 13.59; *p* = 0.02), need for mechanical ventilation (OR = 4.48; CI = 1.82, 11.02; *p* = 0.001), and use of vasoactive drugs (OR = 4.53; CI = 1.84, 11.16; *p* = 0.001). The other variables tested with logistic regression were not associated with mortality.

**Table 4:** Independent risk factors for mortality (age > 70 years)

Variables	OR(CI)	*p*
Oliguria	1.95 (0.86-4.42)	0.10
Diuretics	0.75 (0.34-1.63)	0.47
Dialysis	0.58 (0.19-1.75)	0.33
Septic shock	3.97 (1.15-13.59)	0.02
Mechanical ventilation	4.48 (1.82-11.02)	0.001
Vasoactive drugs	4.53 (1.84-11.16)	0.001

OR - odds ratio, CI confidence interval.

In regards to PSM (Table 5), in the group of 171 patients not offered dialysis PSM found 54 matches (controls not submitted to dialysis) for the 57 patients aged 70+ years who underwent dialysis. After the groups were matched (dialysis and non-dialysis patients) for the main variables correlated with death, no significant difference in mortality was observed between the patients offered dialysis and the individuals treated conservatively.

**Table 5:** Distribution of variables for patients aged 70+ years based on prescription of dialysis during ICU stay, before and after PSM

	Before Matching			After Matching		
Variables	Non-dialysis (n = 167)	Dialysis (n = 57)	*p*	Non-dialysis (n = 54)	Dialysis (n = 57)	*p*
Age (years)	80.8 ± 6.9	78.7 ± 6.2	0.035	79 ± 5.6	78.7 ± 6.2	0.755
Sex			0.760			0.385
Male	84 (36.8)	30 (13.2)		23 (20.7)	30 (27.0)	
Female	87 (38.2)	27 (11.8)		31 (27.9)	27 (24.3)	
KDIGO			0.085			0.797
KDIGO1	49 (21.5)	11 (4.8)		8 (7.2)	11 (9.9)	
KDIGO2	46 (20.2)	11 (4.8)		10 (9.0)	11 (9.9)	
KDIGO3	76 (33.3)	35 (15.4)		36 (32.4)	35 (31.5)	
Charlson	7.4 ± 2.0	7.8 ± 1.9	0.184	7.4 ± 2.0	7.8 ± 1.9	0.283
Delta Nephro	3.6 ± 6.6	4.4 ± 8.1	0.458	3.6 ± 5.8	4.4 ± 8.08	0.594
Higher BUN	156 ± 65.4	228 ± 81.8	< 0.001	154 ± 56	228 ± 81.8	< 0.001
Higher K	5.2 ± 1.2	6.3 ± 3.8	0.001	5.23 ± 0.9	6.3 ± 3.8	0.046
Lower Hb	8.7 ± 2.1	7.5 ± 1.6	< 0.001	8.4 ± 1.9	7.5 ± 1.6	0.005
Higher Leukocyte count	21597.4 ± 21474.7	29221.9 ± 11433.8	0.011	25678.3 ± 21646.4	29221.9 ± 11433.8	0.280
Lower Platelet count	128874.8 ± 79029.3	97491.2 ± 63476.1	0.003	114918.5 ± 61732.6	97491.2 ± 63476.1	0.146
Oliguria	86 (37.7)	49 (21.5)	< 0.001	45 (40.5)	49 (44.1)	0.904
Septic shock	32 (14.0)	20 (8.8)	0.018	18 (16.2)	20 (18.0)	1.000
Diuretics	89 (39.0)	40 (17.5)	0.025	32 (28.8)	40 (36.0)	0.315
VAD	64 (28.1)	37 (16.2)	0.001	32 (28.8)	37 (33.3)	0.676
MV	118 (51.8)	55 (24.1)	< 0.001	51 (45.9)	55 (49.5)	0.951
Mortality	98 (43.0)	47 (20.6)	0.001	42 (37.8)	47 (42.3)	0.704

PSM: propensity score matching; VAD: vasoactive drugs; MV: mechanical ventilation.

## DISCUSSION

This study comprised data taken from a population of elderly individuals, most with severe forms of AKI (48% on KDIGO 3) and critically ill (71.7% on mechanical ventilation and 48% taking vasoactive drugs).

In recent years, there has been discussion as to whether advanced life support (mechanical ventilation, vasoactive drugs, dialysis) should be offered to elderly patients with multiple comorbidities or organ dysfunction. The reasons for the debate include the elevated death rates observed in patients fitting this profile despite the availability of advanced life support and dialysis.^11^^-^ 13 Furthermore, there is little data in the literature - and particularly a lack of publications reflecting the situation in Brazil - on the impact of dialysis on elderly patients on intensive care.

Although dialysis may help patients maintain homeostasis until the recovery of renal function, the procedure has its risks (complications related to the vascular access, hemodynamic instability, bleeding caused by anticoagulant therapy). This is why the impact of dialysis on the death of critically ill patients has been discussed in recent years, with authors reporting worse outcomes in individuals offered dialysis than in patients managed conservatively, even when they were controlled for other factors related to mortality.^14^^,^ 15

In this study, the patients aged 70-80 and 80+ years shared many characteristics (comorbidity index, need for mechanical ventilation and vasoactive drugs) and were analyzed together for the risk factors leading to death and for the impact of dialysis on their outcomes. As shown in previous studies, need for mechanical ventilation, use of vasoactive drugs, and septic shock were independent risk factors for death in our group of patients.^16^^,^ 17 The study also showed that individuals aged 70+ years categorized as KDIGO 3 on mechanical ventilation and vasoactive drugs had a death rate of 93.5%, while 100% of the patients aged 80+ fitting the same profile died in spite of dialysis. With this data in mind and in order to analyze the influence of dialysis on individuals aged 70+ years, the patients were divided into two groups based on whether they underwent dialysis; an attempt was also made to match patients for the main variables correlated with death. Patients were matched based on Propensity Score Matching, a statistical method often used in retrospective clinical trials for its effectiveness in controlling for variables between analyzed groups.^18^ In PSM, patients on dialysis and individuals treated conservatively were matched for variables independently associated with mortality in our sample (Tables 4 and 5). After controlling for these variables, similar death rates were observed between patients treated conservatively and individuals offered dialysis. This finding suggested that dialysis did not have any impact on the mortality of this subgroup of patients (critically ill patients aged 70+ years).

ICUs are filled with elderly patients, and everyday physicians are faced with situations in which dialysis might be prescribed if only workup parameters were considered. However, many patients are aged 70+ years and have decreased renal functional reserve and other organ dysfunctions as a result of senescence; and in addition to AKI, many need invasive ventilation and are hemodynamically unstable.

It should be noted that very few patients aged 80+ years were prescribed dialysis, even when they were categorized as KDIGO 3, when compared to individuals in the other age groups (28% *vs*. 46.2% of the individuals aged 60-70 years and 41.8% of the patients aged 70-80 years) as previously reported in the literature.^19^ A probable explanation for this finding is the higher occurrence of comorbidities in this group or the desire of the patients or their families not to submit to invasive procedures at the end of life. However, since the families of non-dialysis patients were not interviewed, this explanation is merely the fruit of speculation. The lack of clinical conditions to perform dialysis might have been the actual reason for it.

Considering that previous epidemiological studies reported elevated death rates among critically ill elderly patients with AKI, in some countries the idea of offering time-limited trials of dialysis^20^ is gaining strength. In it, dialysis is started and the patient is observed for a few days for global clinical progression (hemodynamic patterns, renal function, and other organ dysfunctions). When none of the parameters improve in one or two weeks, the procedure is suspended. It should be noted that in the absence of national consensus statements on the matter, the decision to suspend dialysis must be made with the agreement of the patients or their families, the assisting physician, and the nephrology and intensive care teams.

A relevant point derived from the findings published in this study is that the information discussed here may provide additional input to patient families and physicians and further inform their discussions based on recent scientific evidence on the degree of support that should be offered to elderly patients on intensive care, an ever present reality in ICUs all over the world.^21^ These discussions help decrease the number of cases of dysthanasia, in which life is prolonged without consideration to quality of life. Another relevant fact is that, to our knowledge, this was the first Brazilian study to assess the impact of dialysis on the care of elderly individuals controlling for variables correlated with death using Propensity Score Matching.

Although the hospital in which data was collected is a reference center in our State, one of the limitations of this study was the fact that it only enrolled patients from one center. Therefore, its findings cannot be generalized to other populations. Other limitations include the retrospective nature of the study and the small size of the included patient sample.

## CONCLUSION

A low proportion of patients aged 80+ years underwent dialysis, possibly on account of external factors such as the desire of the patients or their families. The main risk factors for death were septic shock, use of vasoactive drugs, and mechanical ventilation. In individuals aged 70+ years, dialysis did not reduce mortality.
